# Oral Surgery Clinic

**Published:** 1890-06

**Authors:** 


### ORAL SURGERY CLINIC.—MERCY HOSPITAL, CHICAGO.

SERVICE OF PROFESSOR JOHN S. MARSHALL, M.D., BEFORE THE CLASS OF THE UNIVERSITY DENTAL COLLEGE.[1]

The patient before you, gentlemen, is thirty-eight years old, and the mother of five little children. She is here for treatment of extensive necrosis of the inferior maxillary bone. The odor emitted from the dead bone is almost intolerable, as no doubt your olfactories have already discovered. The patient brings a letter from her doctor, which details briefly the history of the case, and is as follows:

Eleven months ago patient was suffering from an abscessed bicuspid tooth in the inferior maxilla of the right side, which had progressed so far as to open externally under the jaw. Poultices were applied, and later the tooth was extracted. Patient was at this time in the eighth month of gestation.

The external opening soon after closed and remained so for a few weeks, when it again began to discharge. Necrosis of the jaw was then diagnosticated and several loose teeth upon the right side were extracted. The disease has steadily progressed until it is feared the entire bone is involved from the temporo-maxillary articulation of the right side to a corresponding point upon the other. No operation had been permitted by the patient save the extraction of the teeth as they became loosened; the only one now remaining is the wisdom tooth of the right side. The patient is now well anæsthetized, but we shall have to proceed carefully on account of her extreme weakness. Her pulse yesterday was 130; temperature, 100°; respiration, 24.

Stimulants have been administered to brace her for the operation.

The bandages are now removed, and you will discover that the soft tissues, including the chin and anterior portion of the floor of the mouth, the skin of the neck from the angle of the jaw upon one side to a like point upon the other and downward to the cricoid cartilage, and all the muscles having attachment to the inner surface and lower border of the jaw, have sloughed from the bone, so that nearly the entire hand can be passed through the opening

[1] Reported for the INTERNATIONAL DENTAL JOURNAL.

into the mouth. The submaxillary glands are also exposed. Articulate speech is impossible, and all her communications have been made for some weeks by writing.

We will now proceed to examine the extent of the necrosed bone. On the right side it has progressed seemingly to about half an inch above the angle and extends to the temporo-maxillary articulation of the left side. The body of the bone is fractured on a line with the first molar tooth, and it is reasonable to presume this was a natural separation of the sequestrium.

The removal of the inferior maxillary bone, entire or in great part, is not a common operation, and is rarely justifiable except in cases of malignant disease, large tumors, or when the bone has died *en masse*, as in the case before you. The operation will cause great disfigurement, but when the life of the individual is at stake, this becomes a matter of minor importance.

We are by no means confident of a favorable prognosis in the case, as the patient is very weak and anæmic. The possibility of a fatal issue has been explained to her and to her husband, but, as it is certain that she cannot live long in this condition, they are both anxious for the chance of life which may come from the removal of the diseased jaw.

The usual method for an operation of this magnitude is to make an incision along the lower margin of the jaw and chin and divide the lower lip upon the median line. The first incision is not necessary, on account of the loss of so much tissue beneath the jaw. We therefore divide the lip and secure the vessels; we find no connection of the soft tissues with the bone as far back as the angle upon the right side, and well up on the ramus of the left side; with the periosteotome we strip the remaining portions of the periosteum from the ramus and coronoid process, and the bone is now ready to be disarticulated. We seize the ramus with strong forceps and carry the bone backward and outward, the capsular ligament gives way, and the bone is removed. Hemorrhage is very slight, not a single vessel requiring to be ligatured. This is very fortunate, and quite different from what we anticipated.

Examination of the remaining portion of the jaw of the right side makes it necessary to remove a considerable piece of the ramus; this we will do by dissecting the periosteum from the bone, and with the chain-saw and bone-cutting forceps cut it away. It will be necessary, however, to wait a little before proceeding with the operation, as the pulse and respirations indicate the need of stimulants.

The patient having sufficiently rallied to permit the progress of the operation, we proceed, and find, on cutting away this portion of the ramus, that a large pus-pocket has formed in the cancellated structure of the bone. We will, therefore, scrape away with the curette all diseased bone; this being accomplished, and the wound thoroughly irrigated with a five-per-cent. carbolic-acid solution, we will clip away all gangrenous-looking tissue from the edges of the ulcer and scrape away all necrotic material from the deeper portions, and again thoroughly irrigate the parts. The lower lip must now be united; this we will do by two deep sutures, one at the upper margin and the other at the lower, and two superficial ones in the vermillion border.

A suture must now be passed through the tip of the tongue, and this organ drawn well forward and secured by tying the ends of the suture around the neck to prevent the tongue from falling backward into the fauces and causing suffocation. The wound will now be dressed with iodoform and antiseptic gauze and changed every few hours.

Stimulants and nourishing liquid food will be the after-treatment, and if she is unable to swallow food it must be given by the œsophageal tube. The patient has passed through the operation with less shock than we expected, which leads us to hope that if no complications arise she may possibly recover.

